# Unmasking artifactual links: A reanalysis reveals No direct causal relationship between self-esteem and quality of social relations

**DOI:** 10.1016/j.heliyon.2023.e20397

**Published:** 2023-09-22

**Authors:** Kimmo Sorjonen, Michael Ingre, Bo Melin, Gustav Nilsonne

**Affiliations:** a Department of Clinical Neuroscience, Karolinska Institutet, Stockholm, Sweden; b Department of Gastroenterology and Hepatology, Karolinska University Hospital Huddinge, Karolinska Institutet, Stockholm, Sweden; c Institute for Globally Distributed Open Research and Education (IGDORE), Stockholm, Sweden; d Department of Psychology, Stockholm University, Stockholm, Sweden; e Meta-Research Innovations Center (METRICS), Stanford University, Palo Alto, USA

**Keywords:** Self-esteem, Quality of social relations, Meta-analysis, Artifactual prospective effects, Longitudinal studies, Correlation with measurement errors, Reversion to mediocrity

## Abstract

A meta-analysis conducted by Harris and Orth (2020) found positive prospective cross-lagged effects between quality of social relations and self-esteem in included longitudinal studies. Harris and Orth concluded that the link between self-esteem and quality of social relations is reciprocal and characterized by a positive feedback loop. However, meta-analytic effects were estimated while controlling for a prior measurement of the outcome and such effects are known to be susceptible to artifactual (i.e. spurious) effects due to correlations with measurement errors and reversion to mediocrity. We reanalyzed the same data and found paradoxical effects indicating, simultaneously, both increasing and decreasing effects between self-esteem and social relations. These findings suggest that prospective effects between self-esteem and quality of social relations are artifactual rather than due to a true reciprocal effect. Thus, these findings have important theoretical implications and challenge both the risk regulation model, which posits that self-esteem has a causal effect on quality of social relations, and the sociometer theory, which claims that quality of relations is the cause and self-esteem the effect. The present results prompt further investigation into the underlying mechanisms driving these artifactual associations. Additionally, the study highlights the importance of considering methodological limitations in future meta-analyses to improve the accuracy of causal inferences.

## 1 Introduction

Studies have found positive cross-sectional associations between self-esteem and various indicators of quality of relationships with, for example, one's parents [[1], [2], [3], [4]], peers [5,6], coworkers [7], intimate partner [[8], [9], [10]], and children [1,2]; see Cameron and Granger [11] for meta-analyses. Inevitably, the question arises which of self-esteem and quality of social relations might be the cause and which the effect. The risk regulation model [[12], [13], [14]] suggests that people with low self-esteem may distance themselves from social relations, and judge them as less satisfying, as a protective measure against perceived/anticipated low regard from others. Thus, the risk regulation model posits that self-esteem is the cause and quality of social relations the effect. Contrarily, according to the sociometer theory [[15], [16], [17]], self-esteem can be viewed as a gauge of one's relational value, which would mean that quality of relations is the cause and self-esteem the effect. However, a third possibility is that both self-esteem and quality of social relations are influenced by a third confounding factor. In summary, although most researchers appear to agree on the existence of an association between quality of social relations and self-esteem, consensus about its causal nature is lacking.

Longitudinal analyses may help to establish the direction of influence between associated constructs. However, findings from longitudinal studies of self-esteem and quality of social relations are inconclusive. Some studies have indicated a positive association between self-esteem and subsequent development of quality of social relations [[18], [19], [20]] while others have not [[21], [22], [23]]. Similarly, some studies have found a positive association between quality of social relations and subsequent development of self-esteem [21,23,24] while others have not [19,20]. After conducting a meta-analysis of longitudinal studies, Harris and Orth [25] concluded that the link between quality of social relations and self-esteem is reciprocal and characterized by a positive feedback loop. At the time of writing (2023-07-10), the meta-analysis conducted by Harris and Orth has 137 citations indexed on Web of Science and it is marked as a Highly Cited Paper. Although a detailed review of the citing articles is outside the scope of the present study, we may suspect that many are referring to Harris and Orth as evidence of an increasing effect between self-esteem and quality of social relations. With reference to Harris and Orth [25], researchers have claimed, for example, that “the impact of self-esteem on a variety of health outcomes, including interpersonal relationships, have been demonstrated [26], p.236]”, that “healthy social relationships are an important source of self-esteem [27], p.474]”, and that “previous research has demonstrated a vicious cycle of low self-esteem and compromised social relationships [28], p.903]”. Moreover, there are a number of other meta-analyses that have used the same methodology as in Harris and Orth, i.e. where controlled cross-lagged regression effects are estimated from zero-order correlations [e.g. [[29], [30], [31], [32]]].

In their meta-analysis, Harris and Orth [25] estimated the effect of self-esteem on subsequent quality of relations while controlling for initial quality of relations, and vice versa. It is recognized that such controlled effects can be artifactual (i.e. spurious) due to a combination of an association between the predictor and true value on the outcome, less than perfect reliability in the measurement of the outcome, and reversion to mediocrity [[33], [34], [35], [36]]. As an example, assume a cross-sectional correlation (positive) between quality of social relations and self-esteem. This correlation may, for instance, be caused by a confounding impact by a negativity/positivity trait. Consequently, if persons with different self-esteem scores have the same initial quality of relations score, we may suspect a negative association between measured self-esteem and errors in the measurement of quality of relations. This would mean that individuals with a high self-esteem score tended to obtain a low quality of relations score compared with their true quality of relations value, i.e. they have a negative measurement error. Conversely, individuals with a low self-esteem value tended to obtain a high quality of relations score compared with their true quality of relations value, i.e. they have a positive measurement error. However, measurement errors tend to change toward an average value of zero from one measurement to the next and we should, consequently, predict a more positive, but artifactual, change in quality of relations to a subsequent measurement for individuals with a high self-esteem value compared with individuals with the same initial quality of relations value but with a lower self-esteem value. Interestingly, reversion to mediocrity is independent of the direction of time, meaning that we should predict a positive, but still artifactual, effect of self-esteem on the initial quality of relations score when controlling for the subsequent quality of relations score. It should be noted that while the first of these artifactual positive effects would suggest an increasing effect of self-esteem on quality of relations the second positive effect would suggest, simultaneously and paradoxically, a decreasing effect.

The aim of this study was to investigate the association between quality of social relations and self-esteem by re-calculating effects in the studies that were included in the meta-analysis conducted by Harris and Orth [25]. Three different effects of initial self-esteem on quality of social relations, and vice versa, were calculated. Distinct predictions could be made about these effects depending on if they were due to a true increasing effect or artifactual (see the Method section). The present study was intended to fill the gap between the conclusion by Harris and Orth [25], that there is a reciprocal link between self-esteem and quality of social relations, and knowledge that cross-lagged panel analyses can give biased results. Previous longitudinal studies have yielded inconclusive results regarding the causal relationship between self-esteem and social relations. This underscores the need for a rigorous reanalysis to shed light on the true nature of the association. The meta-analysis by Harris and Orth has gained substantial attention and influence in the field, and has been interpreted as supporting reciprocal effects. However, as will become apparent, the current reanalysis challenges these prevailing assumptions and reveals potential artifactual associations, leading to a more nuanced understanding of the underlying dynamics and motivating new research directions. The research question of the present study was: “May the meta-analytic reciprocal prospective effects between self-esteem and quality of social relations presented by Harris and Orth have been artifactual?“. Our hypothesis was that findings by Harris and Orth may have been artifactual due to associations with measurement errors and reversion to mediocrity and, in extension, that both the risk regulation model and the sociometer theory may be wrong.

## 2 Method

### 2.1 Data extraction

See Harris and Orth [25] for more complete information on how studies were selected, tests of bias in publication, and description of the included studies. A list of included studies with abstracts is obtainable from the Open Science Framework at https://osf.io/sm6ex/. Reading the abstracts would give a better understanding of the type of research the meta-analysis by Harris and Orth, and consequently our reanalysis, was built upon. Available at the same location at the Open Science Framework are also data files, with and without comments, and forest plots showing extracted correlations and estimated regression effects for the included studies. Briefly, Harris and Orth extracted zero-order correlations from 42 studies, with data from 53 samples in total. Data in the included studies were collected between 1979 and 2011. Mean age of participants was 21.0 years (*SD* = 15.3, range = 4.1–76.6). Mean portion of female participants was 54% (*SD* = 31%, range = 0–100%).

Of the 53 included samples, 34 used the Rosenberg Self-Esteem scale [37], 10 used one of the Harter Self-Perception Profiles [38], 3 used one of the Marsh Self-Description Questionnaires [39], and 6 used other measures of self-esteem. Of the 53 included samples, 43 used self-reported measures of the quality of social relationships and 10 used informant-reported, observer-reported or some combination of reporters. In 16 studies respondents were asked about their relationship with parents, in 10 about peers, in 5 about romantic partners, in 13 about general others, and in 9 some other relationships. Items used to measure quality of relationships could ask, for example, about perceived acceptance, connectedness, warmth, support, closeness, relationship satisfaction, and low conflict. Harris and Orth [25] did not declare whether they followed PRISMA guidelines. However, it is important to note that whether Harris and Orth followed PRISMA guidelines or not is not material for how the present reanalysis was conducted nor the conclusions it resulted in. As an analogy, how the apples were picked is not material when assessing if somebody has correctly counted the number of apples.

We extracted, when available, all six correlations between self-esteem and quality of relations measured at two occasions from the studies included by Harris and Orth. If more than one measure of self-esteem or quality of relations was reported from the same sample of participants, we calculated a mean of the correlations (after first Fisher's z-transforming them and then inverse back to a non-transformed mean correlation). In several cases it was unclear if the included studies reported analytic sample sizes, initial sample sizes, or the sample size at some other wave of measurement (see our commented data file at the Open Science Framework). As the present study was a reanalysis of Harris and Orth [25], in the end we decided to use the sample sizes reported by them.

### 2.2 Statistical analysis

Eq. (1) gives the expected effect of a predictor P1 on an outcome Q2 when controlling for Q1 [40]. The symbols in Eq. (1) stand for: *E|β*_*P1,Q2.Q1*_*|* = expected standardized regression effect of P1 on Q2 while controlling for Q1; *r*_*P1,Q2*_ = zero-order correlation between P1 and Q2; *r*_*P1,Q1*_ = zero-order correlation between P1 and Q1; *r*_*Q1,Q2*_ = zero-order correlation between Q1 and Q2. Harris and Orth extracted correlations from studies that were included in their meta-analysis and employed Eq. (1) to calculate the effect of self-esteem at T1 (i.e. time 1) on quality of social relations at T2 (i.e time 2) when controlling for quality of relations at T1. This estimation required three zero-order correlations, namely between self-esteem at T1 and quality of relations at T2, between self-esteem at T1 and quality of relations at T1, and between quality of relations at T1 and quality of relations at T2. As an example, these three correlations were equal to 0.43, 0.52, and 0.59, respectively, in a study by Gest et al. [41]. Entering these correlations into Eq. (1), we obtain an estimated cross-lagged effect of self-esteem at T1 on quality of relations at T2 while controlling for quality of relations at T1 equal to 0.17 (Eq. (2)). Eq. (1) was also used to calculate the effect of quality of social relations at T1 on self-esteem at T2 when controlling for self-esteem at T1. This estimation also required three zero-order correlations, namely between quality of relations at T1 and self-esteem at T2, between quality of relations at T1 and self-esteem at T1, and between self-esteem at T1 and self-esteem at T2. As an example, these three correlations were equal to 0.42, 0.52, and 0.56, respectively, in a study by Gest et al. [41]. Entering these correlations into Eq. (1), we obtain an estimated cross-lagged effect of quality of relations at T1 on self-esteem at T2 when controlling for self-esteem at T1 equal to 0.18 (Eq. (3)). Both a hypothesis of genuine (i.e. non-artifactual) reciprocal effects, and a hypothesis of artifactual effects due to correlation with measurement errors and reversion to mediocrity, predicted positive controlled effects of self-esteem at T1 on quality of relations at T2 when controlling for quality of relations at T1, and vice versa.

In addition to the estimations by Harris and Orth, we employed Eq. (1) to calculate the effect of self-esteem at T1 on quality of social relations at T1 when controlling for quality of social relations at T2. This estimation required three zero-order correlations, namely between self-esteem at T1 and quality of relations at T1, between self-esteem at T1 and quality of relations at T2, and between quality of relations at T1 and quality of relations at T2. As an example, these three associations were equal to 0.52, 0.43, and 0.59, respectively, in a study by Gest et al. [41]. Entering these correlations into Eq. (1), we obtain a calculated effect of self-esteem at T1 on quality of relations at T1 when controlling for quality of relations at T2 equal to 0.33 (Eq. (4)). We also employed Eq. (1) to calculate the effect of quality of relations at T1 on self-esteem at T1 when controlling for self-esteem at T2. This estimation also required three zero-order correlations, namely between quality of relations at T1 and self-esteem at T1, between quality of relations at T1 and self-esteem at T2, and between self-esteem at T1 and self-esteem at T2. As an example, these three correlations were equal to 0.52, 0.42, and 0.56, respectively, in a study by Gest et al. [41]. Entering these correlations into Eq. (1), we obtain a calculated effect of quality of relations at T1 on self-esteem at T1 when controlling for self-esteem at T2 equal to 0.35 (Eq. (5)). Here, a hypothesis of genuine reciprocal effects forecasted negative effects, meaning that given the same quality of social relations (self-esteem) at T2, individuals with higher self-esteem (quality of social relations) at T1 would tend to have had lower quality of social relation (self-esteem) at T1 and had, therefore, experienced a larger increase in quality of social relations (self-esteem) between the measurements compared with individuals with the same quality of social relations (self-esteem) at T2 but with lower self-esteem (quality of social relations) at T1. Conversely, a hypothesis of artifactualness forecasted positive effects.

Eq. 1 $$E\left|\beta_{P1,Q2.Q1}\right|=\frac{r_{P1,Q2}-r_{P1,Q1}r_{Q1,Q2}}{1-r_{P1,Q1}^{2}}$$

Eq. 2 $$E\left|\beta_{SE1,QR2.QR1}\right|=\frac{0.43-0.52\times 0.59}{1-0.52^{2}}=0.17$$

Eq. 3 $$E\left|\beta_{QR1,SE2.SE1}\right|=\frac{0.42-0.52\times 0.56}{1-0.52^{2}}=0.18$$

Eq. 4 $$E\left|\beta_{SE1,QR1.QR2}\right|=\frac{0.52-0.43\times 0.59}{1-0.43^{2}}=0.33$$

Eq. 5 $$E\left|\beta_{QR1,SE1.SE2}\right|=\frac{0.52-0.42\times 0.56}{1-0.42^{2}}=0.35$$

Furthermore, we used Eq. (6) [42] to estimate the effect of self-esteem at T1 on change in quality of social relations between the measurements. The symbol on the left-hand side in Eq. (6) stands for the expected standardized regression effect of P1 on the Q2-Q1 difference. The symbols on the right-hand side in Eq. (6) have the same meaning as in Eq. (1) (see above). This estimation required three zero-order correlations, namely between self-esteem at T1 and quality of relations at T1, between self-esteem at T1 and quality of relations at T2, and between quality of relations at T1 and quality of relations at T2. As an example, these three associations were equal to 0.52, 0.43, and 0.59, respectively, in a study by Gest et al. [41]. Entering these correlations into Eq. (6), we obtain an estimated effect of self-esteem at T1 on change in quality of relations between T1 and T2 equal to −0.10 (Eq. (7)). We also employed Eq. (6) to calculate the effect of quality of relations at T1 on change in self-esteem between the measurements. This estimation also required three zero-order correlations, namely between quality of relations at T1 and self-esteem at T1, between quality of relations at T1 and self-esteem at T2, and between self-esteem at T1 and self-esteem at T2. As an example, these three correlations were equal to 0.52, 0.42, and 0.56, respectively, in a study by Gest et al. [41]. Entering these correlations into Eq. (6), we obtain an estimated effect of quality of relations at T1 on change in self-esteem between T1 and T2 equal to −0.11 (Eq. (8)). Here, a hypothesis of genuine reciprocal effects forecasted positive effects. Contrarily, a hypothesis of artifactualness forecasted either negative effects (if the contemporaneous association at T1 was stronger than the cross-lagged associations) or effects close to zero (if the contemporaneous association between self-esteem and quality of social relations at T1 was approximately as strong as the cross-lagged associations). We believe that the first alternative, resulting in negative effects, is more likely. Table 1 summarizes the forecasts of a hypothesis of genuine reciprocal effects and of artifactual effects due to associations with measurement errors and reversion to mediocrity.

Eq. 6 $$E\left|\beta_{P1,Q2-Q1}\right|=\frac{r_{P1,Q2}-r_{P1,Q1}}{\sqrt{2\left(1-r_{Q1,Q2}\right)}}$$

Eq. 7 $$E\left|\beta_{SE1,QR2-QR1}\right|=\frac{0.43-0.52}{\sqrt{2\left(1-0.59\right)}}=-0.10$$

Eq. 8 $$E\left|\beta_{QR1,SE2-SE1}\right|=\frac{0.42-0.52}{\sqrt{2\left(1-0.56\right)}}=-0.11$$

**Table 1:** Predicted sign of effects between self-esteem and quality of social relations according to a hypothesis of genuine reciprocal effects and a hypothesis of artifactualness.

Effect	Reciprocal	Artifactualness
β(SE1,QR2.QR1)	Positive	Positive
β(QR1,SE2.SE1)	Positive	Positive
β(SE1,QR1.QR2)	Negative	Positive
β(QR1,SE1.SE2)	Negative	Positive
β(SE1,QR2-QR1)	Positive	Zero or negative
β(QR1,SE2−SE1)	Positive	Zero or negative

Note: SE = self-esteem; QR = quality of relations; 1 = time 1; 2 = time 2; the variables are given in the order predictor, outcome, and covariate.

We used Eq. (1) in the present study because we wanted to estimate the effect of self-esteem at T1 on quality of social relations at T2 when controlling for quality of social relations at T1, and vice versa. Moreover, we employed Eq. (1) because we wanted to estimate the effect of self-esteem at T1 on quality of social relations at T1 when controlling for quality of social relations at T2, and vice versa. Furthermore, Eq. (1) was used in the challenged study by Harris and Orth [25]. Eq. (1) has been used in other meta-analyses employing the same methodology as in Harris and Orth, e.g. in meta-analyses of prospective effects between academic self-concept and academic achievement [29], self-esteem and work experiences [30], peer-influence [31], and social support and post-traumatic stress disorder [32]. We used Eq. (6) in the present study because we wanted to estimate the uncontrolled (i.e. crude) effect of self-esteem on change in quality of social relations between T1 and T2, and vice versa. Eq. (6) has been used in some previous studies [e.g. [43,44]].

### 2.3 Meta-analysis

We performed a multilevel meta-analysis with random effects for each of the six zero-order correlations, as well as for each of the six effects in Table 1. Fisher's z-transformed regression effects (standardized) were used in the analyses, but these were reversed back to ordinary (i.e. non-transformed) effects for presentations. For studies including effects from multiple samples, the effects were combined with a multilevel approach. In the next step, a meta-analytic effect (random), with a confidence interval (95%), was estimated across the independent effect sizes. As the present study was a reanalysis of the meta-analysis conducted by Harris and Orth [25] rather than an independent meta-analysis, we did not assess potential bias in the included studies. Harris and Orth reported no evidence for publication bias. Analyses were performed with R 4.1.3 statistical software [45] using the metafor package [46].

Differently from Harris and Orth [25], we limited our analyses to regression effects estimated from zero-order correlations with Eq. (1) and Eq. (6). We did not include controlled prospective effects between quality of relations and self-esteem that were reported without presenting zero-order correlations. We made this choice in order to be consistent across the different models. The effects between concurrent measurements of quality of relations and self-esteem when controlling for subsequent measures, or between one of the variables and change in the other variable, were, as far as we could judge, not presented in any of the studies. Consequently, our meta-analyses were based on data from fewer samples and with a smaller total sample size than in Harris and Orth [25]. Compared with 35 samples and 21,995 participants for the prospective effect of self-esteem and 48 samples and 46,231 participants for the prospective effect of quality of relations in Harris and Orth, our analyses were based on data from 27 samples and 12,589 participants and from 37 samples and 21,077 participants, respectively. Reduced number of samples and total number of participants may have had a negative impact on the statistical power of our analyses compared with the analyses by Harris and Orth. However, our estimations of the controlled prospective effects (0.081 and 0.073 for the effect of self-esteem on quality of relations and of quality of relations on self-esteem, respectively) were very similar to those reported by Harris and Orth (0.08 for both effects) and statistically significant. Furthermore, as will become apparent, findings from our alternative models were also statistically significant, but with the wrong sign compared with forecasts by a hypothesis of genuine reciprocal effects. With higher statistical power the effects would be expected to have been even more statistically significant but not to have the opposite sign, i.e. they would still suggest that prospective effects between self-esteem and quality of social relations were artifactual due to associations with measurement errors and reversion to mediocrity. This means that the reduced sample size compared with Harris and Orth should not have affected our conclusions.

An analysis script, forest plots, a list of studies included in the meta-analyses, and data are obtainable from the Open Science Framework at https://osf.io/sm6ex/. The forest plots show effect sizes and confidence intervals for all included studies, as well as estimated meta-analytic effects. The analysis script contains the R code used for all analyses and is annotated.

## 3 Results

Meta-analytic estimates of associations between quality of social relations and self-esteem are presented in Table 2. Estimated associations exhibited statistically non-significant and mostly low heterogeneity, as calculated by *I*^*2*^, which calculates proportion (in %) of variance across effects that can be attributed to heterogeneity rather than random variation, and Cochran's *Q*. Low heterogeneity suggests that the included effect sizes (after aggregation of effect sizes from the same study) may have come from the same distribution of effect sizes. This increases the likelihood that meta-analytic effects were accurate estimations of true population effects. The Cochran's *Q* statistic has an asymptotic chi-squared distribution and is expected to be equal to the number of included effect sizes minus one (= the degrees of freedom of the test) under the null hypothesis of no significant differences between effect sizes. The *I*^*2*^ statistic can be calculated from the *Q* statistic as *I*^*2*^ = *100* × *(Q – df)/Q*, i.e. it gives the difference between the estimated and expected *Q* statistic in percent.

**Table 2:** Meta-analytic correlations and adjusted regression effects between self-esteem and quality of relations measured at two occasions.

Association	*K*	*NE*	*N*	Estimate (95% CI)	*Q* (df)	*I*^*2*^ (95% CI)
1. *r*(SE1,SE2)	31	40	22379	0.568 (0.512; 0.620)	31 (30)	2.4 (0; 46.2)
2. *r*(SE1,QR1)	33	42	23032	0.284 (0.224; 0.341)	32 (32)	0.0 (0; 41.8)
3. *r*(SE1,QR2)	24	29	15094	0.255 (0.200; 0.309)	22 (23)	0.0 (0; 44.1)
4. *r*(SE2,QR1)	30	38	22099	0.239 (0.183; 0.293)	30 (29)	4.5 (0; 48.9)
5. *r*(SE2,QR2)	25	30	15810	0.341 (0.280; 0.399)	23 (24)	0.0 (0; 45.1)
6. *r*(QR1,QR2)	27	32	15087	0.599 (0.546; 0.647)	26 (26)	0.0 (0; 45.6)
7. β(SE1,QR2.QR1)	23	27	12589	0.081 (0.052; 0.111)	19 (22)	0.0 (0; 41.9)
8. β(SE1,QR1.QR2)	23	27	12589	0.161 (0.109; 0.212)	23 (22)	2.2 (0; 52.6)
9. β(SE1,QR2-QR1)	23	27	12589	−0.050 (−0.084; −0.016)	25 (22)	10.9 (0; 61.6)
10. β(QR1,SE2.SE1)	29	37	21077	0.073 (0.041; 0.105)	36 (28)	22.4 (0; 69.2)
11. β(QR1,SE1.SE2)	29	37	21077	0.171 (0.126; 0.216)	27 (28)	0.0 (0; 42.4)
12. β(QR1,SE2−SE1)	29	37	21077	−0.065 (−0.099; −0.031)	30 (28)	6.3 (0; 53.8)

Note: *K* = number of studies; *NE* = number of effects; *N* = total sample size; *Q* = Cochran's Q; *I*^*2*^ = percentage of variation due to heterogeneity rather than randomness; SE = self-esteem; QR = quality of relations; 1 = time 1; 2 = time 2; the variables are given in the order predictor, outcome, and covariate.

All zero-order correlations were positive and quite substantial (Table 2, rows 1–6). According to Cohen's [47] classification (where correlations between 0.1 and 0.3 are characterized as small, correlations between 0.3 and 0.5 as moderate, and correlations stronger than 0.5 as large), the two auto-regressive correlations were large (*r* = 0.568 for self-esteem and *r* = 0.599 for quality of social relations), while the other correlations were small or moderate. None of the 95% confidence intervals include zero, meaning that the correlations were statistically significant at the conventional cutoff of α = 0.05.

**Fig. 1:**
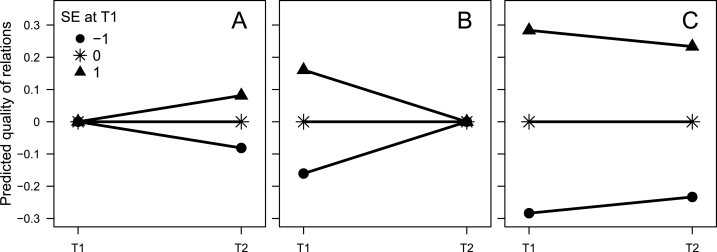
Predicted quality of relations at T2 for individuals with high (1), average (0), and low (−1) self-esteem at T1, respectively, when conditioning on mean quality of relations at T1 (A), at T1 when conditioning on mean quality of relations at T2 (B), and at T1 and T2 when not conditioning on quality of relations at T1 (C).

Self-esteem at T1 was estimated to have a positive, and statistically significant, effect on quality of relations at T2 while controlling for quality of relations at T1 (β = 0.081, 95% CI: 0.052; 0.111, Table 2, row 7). According to benchmarks for controlled cross-lagged effects presented by Orth et al. [48] (0.03 for a small effect, 0.07 for a medium effect, and 0.12 for a large effect), this effect would be characterized as medium. This effect, already shown by Harris and Orth [25], agreed with the risk regulation model [[12], [13], [14]], which suggests that individuals with low self-esteem may distance themselves from social relations, and judge them as less satisfying, as a protective measure against perceived/anticipated low regard from others. Moreover, initial self-esteem was estimated to have a large, and statistically significant, positive effect on initial quality of relations when controlling for subsequent quality of relations (β = 0.161, 95% CI: 0.109; 0.212, row 8). Furthermore, initial self-esteem was estimated to have a small, but statistically significant, negative effect on subsequent change in quality of relations (β = −0.050, 95% CI: −0.084; −0.016, row 9). These effects are illustrated in Fig. 1. It is apparent that high self-esteem at T1 predicted subsequent increase in quality of relations only if the effect was controlled for initial quality of relations (Fig. 1, panel A) but not if the effect was controlled for subsequent quality of relations (Fig. 1, panel B) nor if the effect of self-esteem at T1 on change in quality of relations between T1 and T2 was calculated without adjustment for initial quality of relations (Fig. 1, panel C). A similar description applies to the effect of initial quality of relations on self-esteem (Table 2, rows 10–12), with a medium, and statistically significant, positive effect of quality of relations at T1 on self-esteem at T2 when controlling for self-esteem at T1 (β = 0.073, 95% CI: 0.041; 0.105, row 10), a large, and statistically significant, positive effect of quality of relations at T1 on self-esteem at T1 when controlling for self-esteem at T2 (β = 0.171, 95% CI: 0.126; 0.216, row 11), and a small, but statistically significant, negative effect of quality of relations at T1 on change in self-esteem between T1 and T2 (β = −0.065, 95% CI: −0.099; −0.031, row 12).

Meta-analytic controlled regressions effects (Table 2, rows 7–12) agreed better with a hypothesis of artifactual prospective associations due to associations with measurement errors and reversion to mediocrity than with a hypothesis of genuine increasing prospective effects (see Table 1). A hypothesis of genuine increasing prospective effects was challenged by a positive meta-analytic effect of self-esteem at T1 on quality of social relations at T1 when controlling for quality of social relations at T2 (Table 2, row 8), and vice versa (Table 2, row 11). A hypothesis of genuine increasing prospective effects was also challenged by a negative meta-analytic effect of self-esteem at T1 on change in quality of social relations between T1 and T2 (Table 2, row 9), and vice versa (Table 2, row 12).

## 4 Discussion

### 4.1 Prospective associations between self-esteem and quality of relations

As already shown by Harris and Orth [25], the meta-analysis demonstrated a positive, and statistically significant, effect of self-esteem at T1 on quality of social relations at T2 while controlling for quality of relations at T1, and vice versa. However, the other predictions from a hypothesis of a truly reciprocal effect failed: (1) The effect of self-esteem at T1 on quality of relations at T1 while controlling for quality of relations at T2 was also positive, meaning that those with high initial self-esteem had experienced a higher initial quality of relations and, therefore, a worse development in quality of relations between measurements, compared with individuals with the same quality of relations at T2 but with lower self-esteem at T1. The same was seen when using quality of relations as the predictor and self-esteem as the outcome; (2) Self-esteem at T1 had a weak, but statistically significant, negative effect on change in quality of relations between measurements, and vice versa. This means that a high score on one of these characteristics, compared with a lower score, was predictive of a worse development in the other characteristic. We do not propose that these negative associations should be seen to prove a true decreasing effect. Instead, they probably reflect the fact that associated constructs tend to be more strongly correlated when measured close in time compared to when measured temporally further apart, which tends to result in a negative effect if the constructs are positively correlated (see Eq. (6)).

### 4.2 Alternative explanation: associations with measurement errors and reversion to mediocrity

Due to the failed predictions from a reciprocal effects hypothesis, we propose the alternative explanation that positive prospective controlled effects between quality of relations and self-esteem are artifactual due to associations with measurement errors and reversion to mediocrity. Predictions from this alternative explanation (see Table 1) agreed perfectly with the meta-analytic findings (see Table 2). We may suspect that among individuals with the same measured self-esteem (quality of relations) at T1, individuals with higher measured quality of relations (self-esteem) at T1 have tended to obtain a relatively low value compared with their true self-esteem (quality of relations), i.e. a negative measurement error, while individuals with lower measured quality of relations (self-esteem) at T1 have tended to obtain a relatively high score, i.e. a positive measurement error. Measurement errors tend to reverse toward an average value of zero and we can, consequently, expect those with higher measured quality of relations (self-esteem) at T1 to obtain a higher value on self-esteem (quality of relations) at T2 compared with those with the same value on self-esteem (quality of relations) at T1 but with a lower score on quality of relations (self-esteem) at T1. This interpretation based on associations with measurement errors and reversion to mediocrity was corroborated by the positive effect of self-esteem at T1 on quality of relations at T1 when controlling for quality of relations at T2, and vice versa. Hence, the results from the meta-analysis conducted by Harris and Orth [25], re-analyzed here, should not be seen to verify anything besides a positive correlation between measures of self-esteem and quality of social relations.

**Fig. 2:**
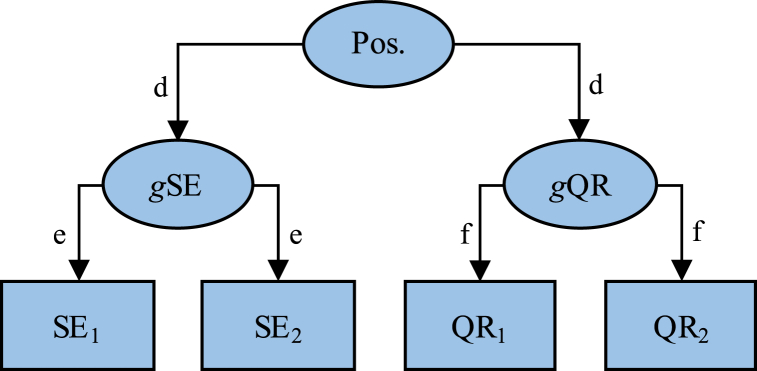
A hypothetical data producing model where general positivity (Pos.) affects trait-like (i.e. general) self-esteem (*g*SE) and quality of social relations (*g*QR) which, in turn, affect measurements at two occasions. The model does not contain any genuine prospective effects between quality of relations and self-esteem, but we may still expect artifactual effects of self-esteem at T1 on quality of relations at T2 while controlling for quality of relations at T1, and vice versa. A more complete description is available in the text.

Moreover, the association between quality of social relations and self-esteem may be caused by a confounding influence by some other variable, e.g. some negativity/positivity trait, or even just some general tendency to respond positively/negatively to items in questionnaires. For example, if data were to have been produced without any genuine prospective effects between quality of social relations and self-esteem, as in Fig. 2, the expected correlation between SE_1_ and QR_2_ and between SE_1_ and QR_1_ would equal *e* × *d* × *d* × *f* = *d*^*2*^*ef* and the anticipated association between QR_1_ and QR_2_ would equal *f* × *f* = *f*^*2*^. Entering these expected correlations into Eq. (1), we obtain:

Eq. 9 $$E\left|\beta_{SE1,QR2.QR1}\right|=\frac{d^{2}ef-d^{2}ef∙f^{2}}{1-{\left(d^{2}ef\right)}^{2}}=\frac{d^{2}ef\left(1-f^{2}\right)}{1-{\left(d^{2}ef\right)}^{2}}$$

Assuming that all three effects (standardized) *d*, *e*, and *f* in Fig. 2 are above zero (>0) but less than one (<1), both the denominator and the numerator in Eq. (9) would also be above zero (>0) but less than one (<1) and the anticipated effect (standardized) of self-esteem at T1 on quality of relations at T2 when controlling for quality of relations at T1 would be positive (>0), even if data had been produced by a model lacking a genuine increasing prospective effect of self-esteem on quality of relations. This shows that presence of a true prospective effect is not necessary for an estimated prospective effect. The same argument holds for an effect of quality of relations at T1 on self-esteem at T2 when controlling for self-esteem at T1.

In summary, as the present findings suggested that prospective effects between quality of social relations and self-esteem may have been artifactual rather than truly increasing, the results from the present reanalysis lend no support to, and cast doubt on, theories and hypotheses about prospective effects between quality of social relations and self-esteem, e.g. the risk regulation model [[12], [13], [14]] or the sociometer theory [[15], [16], [17]]. The present findings could even be seen to suggest that the whole research field of self-esteem and quality of social relations, and theories and hypotheses therein, may have been built on an unstable foundation of spurious associations and statistical artifacts.

### 4.3 Alternatives to cross-lagged panel models

As mentioned in the introduction, limitations and bias of the cross-lagged panel model, applied on data from two waves of measurement, are recognized. It has been argued that stronger inference about causal effects between constructs is allowed if longitudinal data is analyzed with alternative statistical methods such as latent growth curve modeling [49,50] or random-intercept cross-lagged panel models [51,52]. See, for instance, Usami et al. [53], Orth et al. [54], and Lüdtke and Robitzsch [55] for reviews of methods that can be employed to analyze longitudinal data. A presumed advantage of random-intercept cross-lagged panel analyses compared with traditional cross-lagged panel analyses is an ability to control for individuals' stable trait-like levels on the analyzed constructs. An advantage of latent growth curve analyses compared with traditional cross-lagged panel analyses is an ability to differentiate between individuals’ longitudinal trajectories on the analyzed constructs and temporary changes due to random fluctuations around those trajectories. However, these extended methods typically require data from more than two waves of measurement. Moreover, these methods are versions of regression models and an often overlooked assumption in regression analysis is that all possible confounders are included in the analyses and measured with perfect reliability, i.e. without error [40]. Situations where researchers can guarantee that these assumptions are met are probably very rare.

Therefore, we believe that it is extremely hard, perhaps not even possible, to infer causality from analyses of observational (i.e. not experimental) data. The present study further underlines this general point. Although this conclusion may seem bleak, we do not believe that illusions about the prospect to infer causality would be of service to the community of researchers. In the specific case of standard cross-lagged panel analyses of data from two waves of measurement, we endorse researchers to estimate inverted effects, i.e. the effect of P1 on Q1 while controlling for Q2 besides the effect of P1 on Q2 when controlling for Q1, and also uncontrolled effects of P1 on the Q2-Q1 difference. Findings that diverge, as in the present study, would indicate artifactualness and carefulness with conclusions would be advised. Conversely, if findings converge, causality hypotheses could be seen as corroborated.

### 4.4 Policy and practice

The present study casts doubt on assumptions of genuine increasing effects between self-esteem and quality of social relations. Consequently, the present findings indicate that interventions to increase self-esteem or quality of social relations as a measure to improve the other might not be the best use of limited resources. Unfortunately, knowing that certain interventions may not be effective does not automatically indicate what alternative interventions are effective. However, this knowledge may increase incentives for more rigorous studies which, in the end, may result in finding more promising interventions. We encourage clinically oriented and knowledgeable researchers and practitioners to come up with ideas on what those interventions might be.

### 4.5 Limitations

Most of the studies that were included in the meta-analysis conducted by Harris and Orth [25], and consequently in the present reanalysis, were conducted in USA or Europe and none were conducted in Africa or South America. Consequently, if the main present finding, that prospective associations between quality of social relations and self-esteem seem to be artifactual, is globally generalizable or not remains an open question. Mean levels of self-esteem seem to vary across cultures, e.g. due to a stronger emphasis placed on fostering a positive self-esteem in Western compared with non-Western cultures [56]. Moreover, self-serving biases tend to be more pronounced in Western compared with non-Western societies [57,58]. At the same time, Westerners tend to be more independent and individualistic while non-Westerners tend to be more interdependent and collectivistic [59]. These differences could be seen to suggest that the dynamic of quality of social relations and self-esteem could vary across different cultures. Extensive discussions on exactly what cultural aspects may affect associations between quality of social relations and self-esteem, and how, is outside the scope of the present study. However, interested readers are recommended to delve into the extensive literature on cross-cultural psychology [e.g. [60,61]].

Instruments and procedures used in the studies included in the meta-analyses might not have been ideal. However, it is important to bear in mind that such characteristics were the same in the calculations of the effect of self-esteem at T1 on quality of relations at T2 when controlling for quality of relations at T1, the effect of self-esteem at T1 on quality of relations at T1 when controlling for quality of relations at T2, and the effect of self-esteem at T1 on change in quality of relations between T1 and T2, and vice versa. Consequently, such sub-optimal characteristics cannot account for why the analyses suggested, incongruently, both increasing and decreasing effects.

We limited our analyses to regression effects estimated from zero-order correlations in two-wave longitudinal data as this was the method employed in the reanalyzed meta-analysis conducted by Harris and Orth [25]. It is conceivable that a study using some different methodology, e.g. analyses of data from several (more than two) measurement waves with, for instance, latent growth curve modeling [49,50] or random-intercept cross-lagged panel models [51,52], would have provided findings more resistant to artifactualness due to associations with measurement errors and reversion to mediocrity, i.e. more robust findings. Consequently, we do not claim to have disproved the existence of genuine (i.e. non-artifactual) effects between quality of social relations and self-esteem once and for all. The main point of the present study is, instead, that the reality of these effects has not been established in the meta-analysis conducted by Harris and Orth. It should be noted that it was not possible for us to conduct analyses with the alternative methods, as the present study was a reanalysis of data used by Harris and Orth and the alternative methods require data from more than two waves of measurement.

As described in the Methods section, unlike Harris and Orth [25], we limited our analyses to regression effects estimated from zero-order correlations with Eq. (1) and Eq. (6). We did not include controlled prospective effects between quality of relations and self-esteem that were reported without presenting zero-order correlations. We made this choice in order to be consistent across the different models. Consequently, our meta-analytic estimations used data from fewer samples and with a smaller total sample size than in Harris and Orth [25]. Reduced total sample size and number of samples may have had a negative impact on the statistical power of our analyses compared with the analyses by Harris and Orth. However, our estimations of the controlled prospective effects (0.081 and 0.073 for the effect of self-esteem on quality of relations and of quality of relations on self-esteem, respectively) were very similar to those reported by Harris and Orth (0.08 for both effects) and statistically significant. Furthermore, findings from our alternative models were also statistically significant (see Table 2), but with the wrong sign compared with predictions by a hypothesis of genuine reciprocal effects (see Table 1). With higher statistical power the effects would be expected to have been even more statistically significant but not to have the opposite sign, i.e. they would still suggest that prospective effects between quality of social relations and self-esteem were artifactual due to associations with measurement errors and reversion to mediocrity. This means that the reduced sample size compared with Harris and Orth should not have affected our conclusions.

## 5 Conclusions

The present reanalysis of a meta-analysis conducted by Harris and Orth [25] revealed incongruent prospective effects between quality of social relations and self-esteem indicating, simultaneously, both increasing and decreasing effects. Consequently, we propose that the observed associations are artifactual due to correlations with measurement errors and reversion to mediocrity rather than, as claimed by Harris and Orth, due to a true reciprocal link. The present findings challenge both the risk regulation model, which posits that self-esteem has a causal effect on quality of social relations, and the sociometer theory, which claims that quality of relations is the cause and self-esteem the effect. Researchers are recommended to verify estimated prospective effects with analyses that can discriminate between true and artifactual effects. The present reanalysis brings fresh insight by challenging claims about causal effects between quality of social relations and self-esteem, in either direction.

The present study casts doubt on assumptions of genuine increasing effects between quality of social relations and self-esteem. Consequently, the present findings suggest that interventions to increase self-esteem or quality of social relations as a measure to improve the other might not be the best use of limited resources. Unfortunately, knowing that certain interventions may not be effective does not automatically indicate what alternative interventions are effective. However, this knowledge may increase incentives for more rigorous studies which, in the end, may result in finding more promising interventions. We encourage clinically oriented and knowledgeable researchers and practitioners to come up with ideas on what those interventions might be.

## Ethical approval

As the present study was a secondary reanalysis of publicly available data, no ethical approval was required.

## Author contribution statement

All authors listed have significantly contributed to the development and the writing of this article.

## Data availability statement

An analysis script, forest plots, a list of studies employed in the meta-analyses, and data are obtainable from the Open Science Framework at https://osf.io/sm6ex/.

## Credit author statement

Kimmo Sorjonen: Conceptualization, Formal analysis, Writing – original draft, Writing – review & editing, Visualization, Project administration.

Michael Ingre: Conceptualization, Writing – review & editing, Supervision.

Bo Melin: Conceptualization, Writing – review & editing, Supervision.

Gustav Nilsonne: Conceptualization, Writing – review & editing, Supervision.

## Declaration of competing interest

The authors declare that they have no known competing financial interests or personal relationships that could have appeared to influence the work reported in this paper.
